# Transposable element profiles reveal cell line identity and loss of heterozygosity in *Drosophila* cell culture

**DOI:** 10.1093/genetics/iyab113

**Published:** 2021-07-15

**Authors:** Shunhua Han, Preston J Basting, Guilherme B Dias, Arthur Luhur, Andrew C Zelhof, Casey M Bergman

**Affiliations:** 1 Department of Genetics and Institute of Bioinformatics, University of Georgia, Athens, GA 30602, USA; 2 Department of Genetics, University of Georgia, Athens, GA 30602, USA; 3 Drosophila Genomics Resource Center, Indiana University, Bloomington, IN 47405, USA; 4 Department of Biology, Indiana University, Bloomington, IN 47405, USA

**Keywords:** *Drosophila*, transposable element, cell culture, cell line authentication, loss of heterozygosity

## Abstract

Cell culture systems allow key insights into biological mechanisms yet suffer from irreproducible outcomes in part because of cross-contamination or mislabeling of cell lines. Cell line misidentification can be mitigated by the use of genotyping protocols, which have been developed for human cell lines but are lacking for many important model species. Here, we leverage the classical observation that transposable elements (TEs) proliferate in cultured *Drosophila* cells to demonstrate that genome-wide TE insertion profiles can reveal the identity and provenance of *Drosophila* cell lines. We identify multiple cases where TE profiles clarify the origin of *Drosophila* cell lines (Sg4, mbn2, and OSS_E) relative to published reports, and also provide evidence that insertions from only a subset of long-terminal repeat retrotransposon families are necessary to mark *Drosophila* cell line identity. We also develop a new bioinformatics approach to detect TE insertions and estimate intra-sample allele frequencies in legacy whole-genome sequencing data (called ngs_te_mapper2), which revealed loss of heterozygosity as a mechanism shaping the unique TE profiles that identify *Drosophila* cell lines. Our work contributes to the general understanding of the forces impacting metazoan genomes as they evolve in cell culture and paves the way for high-throughput protocols that use TE insertions to authenticate cell lines in *Drosophila* and other organisms.

## Teaser

Proper identification of cell lines is a requirement of rigorous research involving cultured cells. Here we develop an innovative strategy to identify cell lines using transposable element profiles and apply this novel strategy one of the most important model species, Drosophila melanogaster. We clarify the provenance of several Drosophila cell lines and show that loss of heterozygosity affects genome evolution in Drosophila cell culture.

## Introduction

Cultured cell lines play essential roles in biological research, providing model systems to support discovery of basic molecular mechanisms and tools to produce biomolecules with medical and industrial relevance. Despite their widespread use, experiments in cultured cells often show non-reproducible outcomes, and increasing the rigor of cell line-based research is a priority of both funders and journals alike (Lorsch *et al.* 2014). One major source of irreproducible research comes from mislabeling or cross-contamination of cell lines (collectively referred to here as “misidentification”), resulting in cells of the wrong type or species being used in a particular study (Defendi *et al.* 1960; Gartler 1967; Nelson-Rees *et al.* 1981; MacLeod *et al.* 1999; Huang *et al.* 2017). As such, substantial effort has been invested into minimizing cell line misidentification through genotyping cell lines, cataloging misidentified lines, standardizing cell line nomenclature, and the use of research resource identifiers (Masters *et al.* 2001; Barallon *et al.* 2010; Capes-Davis *et al.* 2010; Yu *et al*. 2015; Babic *et al.* 2019).

Starting with the first reports on the cell line misidentification problem, a variety of cytological and molecular techniques have been developed to authenticate mammalian cell lines (Defendi *et al.* 1960; Gartler 1967; O’Brien *et al.* 1977; Gilbert *et al.* 1990; Masters *et al.* 2001; Castro *et al.* 2013). These efforts culminated in the development of short tandem repeats (STRs) as a widely used standard to authenticate human cell lines at the molecular level (Masters *et al.* 2001; Barallon *et al.* 2010; Almeida *et al.* 2016). STR-based authentication has mitigated—but not eradicated—the human cell line misidentification problem, in part because of limitations in the stability, measurement, and matching of STRs (Parson *et al.* 2005; American Type Culture Collection Standards Development Organization Workgroup ASN-0002 2010; Yu *et al.* 2015; Horbach and Halffman 2017). More recently, alternative methods for genotyping human cell lines based on single nucleotide polymorphisms (SNPs) have been developed (Castro *et al.* 2013; Liang-Chu *et al.* 2015; Yu *et al.* 2015; Zaaijer *et al*. 2017; Mohammad *et al.* 2019), but these methods have not yet been accepted as standards for cell line authentication in humans (Almeida *et al.* 2016).

For most species beside humans, cell line authentication standards and protocols remain to be established (Almeida *et al.* 2016). For example, no protocols currently exist to authenticate cell lines in the fruit fly *Drosophila melanogaster*, despite the existence of over 150 different cell lines for this model animal system (Luhur *et al.* 2019). As such, no evidence of misidentified *Drosophila* cell lines has been cataloged to date by the International Cell Line Authentication Committee (v10, https://iclac.org/databases/cross-contaminations/). The development of cell line identification protocols and standards for common model organisms like *Drosophila* is an important goal for increasing rigor and reproducibility in bioscience. Achieving this goal for a new species requires an understanding of the genome biology and cell line diversity of that organism, and should ideally take advantage of powerful, cost-effective modern genomic technologies.

Relative to humans, the STR mutation rate is low in *D. melanogaster* (Schug *et al.* 1997) and thus the use of STRs for discriminating different *Drosophila* cell lines is likely to be limited. In contrast, it is well-established that transposable element (TE) insertions are highly polymorphic among individual flies (Charlesworth and Langley 1989) that TE abundance is elevated in *Drosophila* cell lines relative to whole flies (Potter *et al.* 1979; Ilyin *et al.* 1980; Rahman *et al.* 2015), and that TE families amplified in cell culture vary among *Drosophila* cell lines (Echalier 1997; Rahman *et al.* 2015). These properties suggest that TE insertions should be useful markers to discriminate different cell lines established from distinct *D. melanogaster* donor genotypes (*e.g.*, S2 *vs* Kc cells) and possibly also from the same donor genotype, including divergent sublines of the same cell line (*e.g.*, S2 *vs* S2R+ cells) (Echalier and Ohanessian 1969; Schneider 1972; Yanagawa *et al.* 1998). Indeed, previous studies have shown that *D. melanogaster* cell lines have unique TE landscapes and that sublines of the same cell line often share a higher proportion of TE insertions relative to distinct cell lines (Sytnikova *et al.* 2014; Rahman *et al.* 2015).

Here, we show that *Drosophila* cell lines can successfully be clustered and identified on the basis of their genome-wide TE profiles using a combination of publicly available paired-end short-read whole-genome sequencing (WGS) data from the modENCODE project (Lee *et al.* 2014) and new WGS data for eight widely used *Drosophila* cell lines. Our approach reveals the first examples where the reported provenance of *Drosophila* cell lines—Sg4 (Morales *et al.* 2004) and mbn2 (Gateff *et al.* 1980)—conflicts with identity inferred from genomic data. Importantly, our TE-based clustering approach also allows us to identify which subset of TE families discriminate the most widely used *Drosophila* cell lines, paving the way for the development of PCR-based genotyping protocols that can be used for cost-effective *Drosophila* cell line identification.

Additionally, we develop a new tool for the detection of TEs in single-end WGS data (called “ngs_te_mapper2”) and integrate our new data with legacy data (Sienski *et al.* 2012; Sytnikova *et al.* 2014) to resolve the history and provenance of the widely used OSS and OSC ovarian cell lines (Niki *et al.* 2006; Saito *et al.* 2009). Using TE-based clustering, we provide evidence that OSS and OSC cell lines can be discriminated on the basis of the *ZAM* retrotransposon family. We propose that the OSS_E subline reported in Sytnikova *et al.* (2014) approximates an ancestral state of the OSC cell line, with contemporary OSC sublines having undergone loss of heterozygosity (LOH) in cell culture from an OSS_E-like state. Together, our results show that TE insertions are a powerful source of genetic markers that can be used for cell line authentication in *Drosophila* and that LOH is an important mechanism driving *Drosophila* cell line genome evolution.

## Materials and methods

### Genome sequencing

Public genome sequencing data for 26 samples of 18 *Drosophila* cell lines were obtained from the modENCODE project (Lee *et al.* 2014). Frozen stocks of eight additional samples from six *Drosophila* cell lines (mbn2, Sg4, ML-DmBG3-c2, ML-DmBG2-c2, OSS, and OSC) were obtained from the *Drosophila* Genomics Resource Center (DGRC), the Gorski lab (Canada’s Michael Smith Genome Sciences Centre, BC Cancer) and the Strand lab (University of Georgia). DNA extractions were performed using Qiagen Blood and Tissue kit (Cat# 69504) for the mbn2 sample from the Strand lab and using the Zymo-Quick kit (Cat# D4068) for all other samples. Purified DNA was analyzed by Qubit and Fragment Analyzer to determine the concentration and size distribution, respectively. Samples were normalized to the same concentration before preparing libraries with the KAPA Hyper Prep Kit (Cat# KK8504). During library prep, DNA was fragmented by acoustic shearing with Covaris E220 Evolution before end repair and A-tailing. Single indices were ligated to DNA fragments. Libraries were purified and cleaned with Solid Phase Reversible Immobilization (SPRI) beads before PCR amplification. Final libraries underwent an additional round of bead cleanup before being assessed by Qubit, qPCR (KAPA Library Quantification Kit Cat# KK4854), and Fragment Analyzer. Libraries were then sequenced in paired-end 150-bp mode on an Illumina NextSeq500 high output flowcell and demultiplexed using bcl2fastq. Metadata, sequencing statistics, and SRA accession numbers for all cell line DNA-seq samples used in this study can be found in Supplementary Table S1.

### Detection of non-reference TE insertions using paired-end sequencing data

Paired-end sequencing data from the modENCODE project (Lee *et al.* 2014) and our study were used as input to seven methods designed to detect non-reference TE insertions in *Drosophila* (Kofler *et al.* 2012, 2016; Linheiro and Bergman 2012; Zhuang *et al*. 2014; Adrion *et al.* 2017; Yu *et al*. 2021) using McClintock (revision 40863acf11052b18afb4cdcd7b1124de48cba397; options: -m “trimgalore, popoolationte, popoolationte2, temp, temp2, teflon, ngs_te_mapper, ngs_te_mapper2”) (Nelson *et al.* 2017). Additionally, we predicted non-reference TE insertions using a version of TIDAL 1.2 (Rahman *et al.* 2015; Yang *et al*. 2021) that was modified to output results in a format compatible with results from McClintock (https://github.com/bergmanlab/TIDAL, revision 2d110b17b3b287dbc1ceb67c87fe171d15095c84). The reference genome for these analyses was comprised of the major chromosome arms from the *D. melanogaster* dm6 assembly (chr2L, chr2R, chr3L, chr3R, chr4, chrM, chrY, and chrX) and the TE library was the Berkeley *Drosophila* Genome Project canonical TE dataset v10.1 (https://github.com/bergmanlab/transposons/blob/master/releases/D_mel_transposon_sequence_set_v10.1.fa; revision f94d53ea10b95c9da99258ac2336ce18871768e9).

Paired-end samples analyzed here vary substantially in read length (50–151 bp) and depth of coverage (5X-136X; Supplementary Table S1). We chose not to normalize input datasets by downsampling to the lowest read length and coverage to avoid reducing the sensitivity of non-reference TE detection methods for higher-quality samples. Using complete samples allowed us to observe that the number of non-reference TE predictions per sample (Supplementary Table S2) showed a strong dependence on read length (Supplementary Figure S1) or coverage (Supplementary Figure S2) for all methods besides TEMP (Zhuang *et al.* 2014). Thus, we used TEMP predictions with default McClintock filtering (retain only 1p1 predictions with >0.1 intra-sample allele frequency cutoff) for the global analysis of the modENCODE-only and expanded (modENCODE plus new samples) datasets. To resolve details of the relationship among mbn2 sublines, we used read length and coverage normalized mbn2 samples with relaxed filtering criteria for TEMP predictions (retain all 1p1/2p/singleton predictions with no intra-sample allele frequency cutoff).

### Detection of non-reference TE insertions using single-end sequencing data

Single-end sequencing data for OSS and OSC cell line samples from two previous studies (Sienski *et al.* 2012; Sytnikova *et al.* 2014) and forward reads from our paired-end samples were used to predict non-reference TE insertions using ngs_te_mapper2 (https://github.com/bergmanlab/ngs_te_mapper2) in McClintock (revision 40863acf11052b18afb4cdcd7b1124de48cba397; options: -m “trimgalore, coverage, ngs_te_mapper2, map_reads”; Nelson *et al.* 2017). ngs_te_mapper2 is a reimplementation of the non-reference TE detection method initially reported in Linheiro and Bergman (2012) that improves speed and sensitivity and has been extended to estimate TE allele frequency (see Supplementary Text for details). Reference genome and TE library files used for McClintock runs on single-end sequencing data were the same as used above for paired-end sequencing data. Because ngs_te_mapper2 detection rates and allele frequency estimates are sensitive to read length and depth of coverage (see Supplementary Text), reads from single-end sequencing data and the forward read of our paired-end sequencing data were normalized by trimming all reads to 100 bp using fastp v0.20.1 (Chen *et al.* 2018) and downsampling to the lowest coverage sample (14X) using seqtk v1.3 (Li 2015).

### Classification of intra-sample TE insertion allele frequency

To predict whether TE insertions within OSS and OSC cell line samples were heterozygous or homozygous, we built a classifier that uses allele frequencies estimated by ngs_te_mapper2 from single-end sequencing data as input. A non-reference TE insertion was predicted to be heterozygous if the intra-sample allele frequency estimated by ngs_te_mapper2 is between 0.25 and 0.75 and predicted to be homozygous if the intra-sample allele frequency is greater than or equal to 0.95. TE insertions with intra-sample allele frequencies outside these ranges were considered unclassified. The classifier was benchmarked using synthetic homozygous and heterozygous WGS datasets created with wgsim v0.3.1-r13 using the ISO1 (dm6) and A4 (GCA_003401745.1; Chakraborty *et al.* 2018) genome assemblies as input. The classifier yields >91% precision using input from the results of ngs_te_mapper2 applied to the simulated datasets (see Supplementary Text for details).

### Identification of orthologous TE insertions

Because positional resolution of non-reference TE predictions is inexact (Nelson *et al.* 2017), we identified a high-quality set of orthologous non-reference TE insertion loci as follows. Genome-wide non-redundant BED files of non-reference TE predictions generated by McClintock were filtered to exclude TEs in low recombination regions using boundaries defined by Cridland *et al.* (2013) lifted over to dm6 coordinates. Normal recombination regions included in our analyses were defined as chrX: 405,967–20,928,973, chr2L: 200,000–20,100,000, chr2R: 6,412,495–25,112,477, chr3L: 100,000–21,906,900, chr3R: 4,774,278–31,974,278. We restricted our analysis to normal recombination regions, since low recombination regions have high reference TE content which reduces the ability to predict non-reference TE insertions (Bergman *et al.* 2006; Manee *et al.* 2018). We also excluded *INE-1* family from our analysis, as this family is reported to be inactive for millions of years (Singh and Petrov 2004; Wang *et al.* 2007). Non-reference TE predictions in high recombination from all samples were then clustered into orthologous loci using BEDtools cluster v2.26.0 enforcing predictions within each cluster to be on the same strand (option -s; Quinlan and Hall 2010). Orthologous loci were then filtered using the following criteria: (1) retain only a single TE family per locus; (2) retain only a single TE prediction per sample per locus; and (3) retain TE predictions only from long-terminal repeat (LTR) retrotransposon, LINE-like retrotransposon or DNA transposon families. For clustering of paired-end samples, we imposed the additional filtering requirement that all clusters include at least sample per locus with a TEMP 1p1 prediction.

### Clustering and identification of cell line samples using TE insertion profiles

Non-reference TE predictions at orthologous loci were then converted to a binary presence/absence matrix in order to cluster cell lines on the basis of their TE insertion profiles. Cell line clustering was performed using Dollo parsimony in PAUP (v4.0a168; Swofford 2003). Dollo parsimony analyses were conducted using heuristic searches with 50 replicates. A hypothetical ancestor carrying the assumed ancestral state for each locus (absence) was included as a root in the analysis (Batzer and Deininger 2002). “DescribeTrees chgList = yes” option was used to assign character state changes to branches in the tree. Node support for the most parsimonious tree was evaluated by integrating 100 bootstrap replicates generated by PAUP using SumTrees (Sukumaran and Holder 2010).

Identification of a cell line sample was performed by adding its TE profile to a binary presence/absence matrix of “primary replicates” of 22 non-redundant *Drosophila* cell line samples and performing cell line clustering using the same approach mentioned above. A phylogenetic tree of the 22 non-redundant primary *Drosophila* cell line samples was used as a backbone topological constraint during a heuristic searches for the most parsimonious tree that included one additional “secondary replicate.” Node support for the most parsimonious tree was evaluated by integrating 100 bootstrap replicates without topological constraints.

### B-allele frequency and copy number analysis

BAM files generated by McClintock were used for variant calling using bcftools v1.9 (Li 2011). Indels were excluded from variant calling, leaving only SNPs in the VCF file. For a given SNP, the B-allele frequency (BAF) was determined as the coverage of reads supporting non-reference allele divided by total coverage at that position using the DP4 field.

BAM files generated by McClintock were also used to generate copy number variant (CNV) profiles for nonoverlapping 10-kb windows of the dm6 genome using Control-FREEC (v11.6; Boeva *et al.* 2012). Windows with less than 85% mappability were excluded from the analysis based on mappability tracks generated by GEM (v1.315 beta; Derrien *et al.* 2012). The baseline ploidy was determined by normalized DNA read density of 10-kb windows following Lee *et al.* (2014). The sex information was determined from relative read density between chromosome X and autosomes. The minimum and maximum expected value of the GC content was set to be 0.3 and 0.45, respectively.

### Clustering of cell line samples based on transcriptomes

Total RNA-sequencing samples for 17 *Drosophila* cell lines with 100-bp paired-end reads were obtained from Stoiber *et al.* (2016) and from the modENCODE *D. melanogaster* transcriptome sequencing project (Brown *et al.* 2014). SRA accession numbers for all cell line RNA-seq samples used in this analysis can be found in Supplementary Table S3. Transcript abundances for protein-coding genes were quantified in unit of transcripts per million using kallisto quant v0.46.2 (Bray *et al.* 2016) using the release 6.32 version of the *D. melanogaster* transcript coding sequences corresponding to Ensembl genes from Ensembl release 103 (http://ftp.ensembl.org/pub/release-103/fasta/drosophila_melanogaster/cds/Drosophila_melanogaster.BDGP6.32.cds.all.fa.gz;Yates *et al*. 2020). Transcript-level abundance estimates were summarized into gene-level abundance estimates using the release 6.32 version of the *D. melanogaster* gene annotation from Ensembl release 103 (http://ftp.ensembl.org/pub/release-103/gtf/drosophila_melanogaster/Drosophila_melanogaster.BDGP6.32.103.gtf.gz) using tximport v1.18.0 (Soneson *et al.* 2015). The summarized gene-level abundance matrix was log-transformed and visualized using the Rtsne package v0.15 (Krijthe 2015) with following parameters: perplexity = 1, theta = 0.0, max_iter = 5000, check_duplicates = FALSE.

## Results and discussion

### Clustering of cell lines using TE insertions reveals rare cases of mismatch with expected provenance

**Figure 1:**
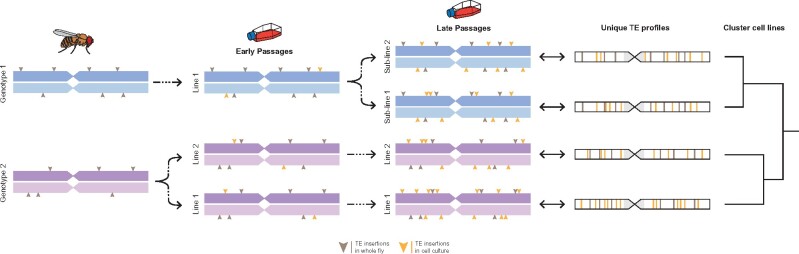
Germline and somatic transposition jointly can create unique TE profiles in *Drosophila* cell line genomes. A homologous pair of chromosomes is shown for two donor fly genotypes used to establish two distinct cell lines. TE profiles initially differ because transposition events in whole flies (gray arrowheads) are maintained at low population frequencies by purifying selection (Charlesworth and Langley 1989). After the establishment of distinct cell lines, ongoing transposition in cell culture (orange arrowheads) leads to increased TE abundance relative to whole flies (Potter *et al.* 1979; Ilyin *et al.* 1980; Rahman *et al.* 2015) and further differentiates TE profiles, both for distinct cell lines derived from the same or different donor genotypes as well as for sublines of the same cell line. Ultimately, these processes lead to unique TE profiles that can identify cell lines and allow them to be clustered based on shared presence or absence of TE insertions at orthologous loci. The model depicts a simplified case of diploidy, when in reality cell culture genomes can have complex genome structure due to polyploidy and segmental aneuploidy.

We reasoned that TE insertions would be favorable genetic markers for cell line identification in *Drosophila* because the joint processes of germline transposition in whole flies and somatic transposition in cell culture together would create unique TE profiles, both for cell lines derived from distinct *D. melanogaster* donor genotypes and for sublines of cells derived from the same original donor genotype (Figure 1). Furthermore, we posited that shared presence or absence of TE insertions at orthologous loci would allow the identity or similarity among cell line samples to be assessed based on a clustering approach.

We initially investigated the possibility of TE-based cell line identification in *Drosophila* using public genome sequences for 26 samples from 18 cell lines generated by the modENCODE project (Lee *et al.* 2014; Supplementary Table S1). Paired-end Illumina WGS sequences were used to predict non-reference TEs using TEMP (Zhuang *et al.* 2014), which showed the least dependence on read length (Supplementary Figure S1) or coverage (Supplementary Figure S2) out of eight non-reference TE detection methods tested on the data used in this study. We clustered cell lines on the basis of their TE profiles using Dollo parsimony, which accounts for the virtually homoplasy-free nature of TE insertions within species (Batzer and Deininger 2002; Ray *et al.* 2006), the ancestral state of TE absence at individual loci (Batzer and Deininger 2002), and false-negative predictions inherent in non-reference TE detection software (Nelson *et al.* 2017; Rishishwar *et al.* 2017; Vendrell-Mir *et al.* 2019). The use of Dollo parsimony for clustering cell line samples also allows ancestral states to be reconstructed, facilitating inference of which TE families diagnostically identify individual cell lines or groups of cell lines. We note that we do not attempt to interpret the clustering relationships among distinct cell lines in an evolutionary context; however, our approach does provide insight into the evolutionary history of clonally evolving sublines established from the same original cell line.

**Figure 2:**
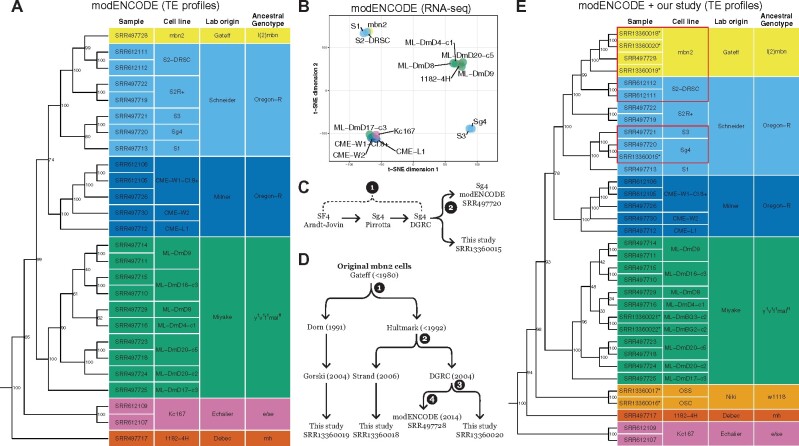
TE insertion profiles cluster *Drosophila* cell lines by lab origin and reveal unexpected placement of the Sg4 and mbn2 cell lines. (A) Clustering of *Drosophila* cell line samples from the modENCODE project was constructed using Dollo parsimony based on non-reference TE insertions. Samples are colorized by the lab origin based on the first publication reporting the original variant of the cell line. Ancestral genotype is based on the *D. melanogaster* stock reported to create the original variant of the cell line. (B) t-SNE visualization of 15 *Drosophila* cell line samples using transcriptomic data in Stoiber *et al.* (2016). Samples are colorized by the lab origin of cell lines. (C) Key events in the history of the Sg4 cell line creation and distribution. (D) Key events in the history of the mbn2 cell line distribution. Node labels in (C) and (D) represent timepoints in the past that potential cell line misidentification events could have occurred. (E) Clustering of *Drosophila* cell line samples from the modENCODE project plus new data reported here (indicated by asterisks in E) was constructed using Dollo parsimony based on non-reference TE insertions. Numbers beside nodes in (A) and (E) indicate percent support based on 100 bootstrap replicates. Red boxes in (E) highlight cases where the reported provenance of *Drosophila* cell lines conflicts with identity inferred from genomic data.

We predicted between 730 and 2579 non-reference TE insertions in euchromatic regions of *Drosophila* cell line samples from the modENCODE project (Supplementary Table S2). As reported previously for human cancer cell lines (Zampella *et al.* 2016), each *Drosophila* cell line sample had a unique profile of TE insertions (Supplementary File S1). The most parsimonious clustering of *Drosophila* cell lines using TE profiles revealed several expected patterns that indicate TE insertions reliably mark the identity of *Drosophila* cell lines (Figure 2A, Supplementary File S2). First, replicate samples of the same cell line cluster most closely with one another with 100% bootstrap support in all seven cases where data are available (S2, S2R+, CME-W1-Cl.8+, ML-DmD9, ML-DmD16-c3, ML-DmD20-c5, and Kc167). Second, different cell lines created in the same lab (presumably from the same ancestral fly genotype) cluster with each other before they cluster with cell lines generated in other labs, or with cells lines having different ancestral genotypes. Third, we observe that divergent sublineages of the same cell line (*i.e.*, S2 and S2R+) cluster closely together (Schneider 1972; Yanagawa *et al.* 1998). We also find weak evidence for clustering of cell lines generated in different labs (Schneider and Milner) that are derived from the same putative ancestral fly stock (Oregon-R). However, we caution against overinterpretation of this result, given previous reports for substantial genetic diversity among common lab stocks like Oregon-R (Rahman *et al.* 2015; Stanley and Kulathinal 2016). Also, cell lines derived from the Schneider and Milner labs have distinct BAF profiles, suggesting different ancestral Oregon-R genotypes (Supplementary Figure S3B).

Overall, clustering patterns based on TE profiles suggest that misidentification is rare among the panel of cell lines sequenced by modENCODE. However, we observed two cases where the similarity of cell lines based on genome-wide TE profiles conflicted with expectations based on reported provenance. First, we unexpectedly found that the Sg4 cell line (originally called Sf4 by its maker Donna Arndt-Jovin) clusters most closely with S3 cells, although the DGRC and FlyBase currently consider Sg4 to be a variant of S2 cells (http://flybase.org/reports/FBrf0205934.html; http://flybase.org/reports/FBtc0000179; https://dgrc.bio.indiana.edu/cells/S2Isolates). More strikingly, we also observed that the mbn2 cell line originally reported by Gateff *et al.* (1980) to be derived from the l(2)mbn stock was placed inside a well-supported cluster containing cell lines (S1, S2, S2R+, S3, and Sg4) generated by Schneider (1972) from an Oregon-R stock. Our clustering of mbn2 cells inside the Schneider cell clade is consistent with a previously unexplained observation that mbn2 cells share an unexpectedly high proportion of TE insertions with both S2 and S2R+ cells (Rahman *et al.* 2015).

Clarification of the provenance of the Sg4 and mbn2 cell lines used by modENCODE is important since many functional genomics resources were generated for these cell lines (Roy *et al.* 2010) and over 125 publications involving these cell lines are curated in FlyBase (Larkin *et al.* 2021). To cross-validate genomic clustering based on TE profiles and to assess potential functional similarity between Sg4↔S3 and mbn2↔S2 cell lines, we clustered cell lines on the basis of their transcriptomes. Transcriptome-based clustering should reveal similarities among cell types rather than genotypes, and thus is not expected to globally match our TE insertion-based clustering. However, both cell type and genotype clustering should support the similarity of pairs of cell lines that are derived from a common ancestral cell line.

Previous transcriptome-based clustering of cell lines based on early whole-genome tiling microarray datasets from the modENCODE project did not reveal similarities among Sg4 and S3 or mbn2 and S2 (Cherbas *et al.* 2011), however, clustering of small RNA-seq data did reveal similarities among these cell lines (Wen *et al.* 2014). Using a consistent batch of poly-A RNA-seq samples from a panel of 15 DGRC cells lines with genome data (Stoiber *et al.* 2016; Supplementary Table S3), we estimated expression levels for protein-coding genes then used T-distributed Stochastic Neighbor Embedding (t-SNE) dimensionality reduction (van der Maaten and Hinton 2008; van der Maaten 2014) to visualize similarity of cell lines based on their gene expression profiles. This analysis revealed that gene expression profiles based on transcriptome data support the clustering of Sg4 with S3 and mbn2 with S2 (Figure 2B). Transcriptome-based clustering of Sg4 with S3 and mbn2 with S2 is also observed in a different batch of RNA-seq samples generated independently by the modENCODE project (Brown *et al.* 2014; Supplementary Figure S4 and Table S3). These results provide replicated transcriptomic support for the clustering of Sg4↔S3 and mbn2↔S2 cell lines revealed by TE profiles and also highlight functional similarities between these pairs of cell lines.

### TE profiles help resolve the provenance of the Sg4 and mbn2 cell lines

To better understand the cause of the surprising patterns of clustering for the Sg4 and mbn2 cell lines in the modeENCODE data, we generated paired-end Illumina WGS sequences for additional samples of Sg4 and mbn2 cells from the DGRC and other sources. In addition, we sequenced several other popular *Drosophila* cell lines (OSS, OSC, ML-DmBG3-c2, and ML-DmBG2-c2) that were not originally sequenced in the modENCODE cell line genome project (Lee *et al.* 2014). To guide sampling and aid the interpretation of the expanded dataset, we reconstructed key events in the history of the Sg4 (Figure 2C) and mbn2 cell lines (Figure 2D). We predicted non-reference TE insertions in these additional samples and then reclustered the expanded dataset using the same methods as the modENCODE-only dataset. The inclusion of additional samples altered some details of the clustering relationships among D-series cell lines generated by the Miyake lab and the position of distantly related cell lines with respect to the root (Kc167 and 1182-4H; Figure 2A *vs* E). However, key aspects of our clustering approach that facilitate cell line identification (replicates clustering most closely, clustering of cell lines from the same lab/ancestral genotype) appear to be robust to the set of cell line samples analyzed.

Clustering TE profiles from this expanded dataset of 34 samples from 22 *Drosophila* cell lines revealed that our resequenced sample of DGRC Sg4 clusters with high support first with the modENCODE sample of DGRC Sg4 then with S3 (Figure 2E). This result confirms the reproducibility of the S3↔Sg4 genomic similarity and rejects the possibility of cell line swap during the modENCODE cell line sequencing project (node 2; Figure 2C). Additional evidence for the similarity of Sg4 and S3 can be observed in their BAF and CNV profiles. All Sg4 and S3 samples are generally devoid of heterozygosity across their entire genomes, including lacking a small patch of heterozygosity at the base of chromosome arm 2L that is present in all S2 or S2R+ samples (Supplementary Figure S3B). All Sg4 and S3 samples also share CNVs on chromosome arms 2L and 3L that are not present in any S2/S2R+ sample (Supplementary Figure S3C). Together, these data support the conclusion that DGRC Sg4 is a variant of the S3 cell line, not the S2 cell line as currently thought. Presently, we are unable to determine where misidentification of Sg4 as a variant of S2 occurred in the provenance chain from initial development of the cell line by the Arndt-Jovin lab to receipt by the DGRC (node 1; Figure 2C). Future analysis of additional Sg4 sublines circulating in the research community (Morales *et al.* 2004; Schwartz *et al.* 2006) will be necessary to establish the timing of this event and if the S3↔Sg4 similarity first observed in the DGRC Sg4 subline is more widespread.

The second case of unexpected clustering we observed in the modENCODE data involving mbn2 and S2 is potentially more surprising and consequential given that these cell lines are reported to be derived from different ancestral genotypes. mbn2 cells were reportedly derived from a stock carrying l(2)mbn on a 2nd chromosome marked with three visible mutations (Gateff 1977; Gateff *et al.* 1980), while S2 cells were derived from a wild-type Oregon-R stock (Schneider 1972). Unfortunately, the l(2)mbn mutation was never characterized at the molecular level, and no fly stocks carrying l(2)mbn currently exist in public stock centers that could be sequenced and compared with the mbn2 cell line. In the absence of external biological resources to verify the identity of an authentic mbn2 cell line, we attempted to infer the timing and extent of the potential mbn2 misidentification event first observed in the modENCODE data by sequencing sublines of mbn2 from DGRC and other sources. We resequenced another sample of the DGRC mbn2 subline, a subline from the Strand lab (University of Georgia) derived from the same donor as the DGRC subline (Hultmark lab, Umeå University), and a subline from the Gorski lab (Canada’s Michael Smith Genome Sciences Centre, BC Cancer) derived from an independent donor (Dorn lab, Johannes Gutenberg-Universität Mainz; Figure 2D). The Hultmark and Dorn labs each report obtaining mbn2 cells directly from the Gateff lab in the early 1990s (Samakovlis *et al.* 1992; Ress *et al.* 2000). This sampling allowed us to infer if potential misidentification occurred during the modENCODE project (node 4), at the DGRC (node 3), in the Hultmark lab (node 2) or in the Gateff lab (node 1; Figure 2D).

Analysis of TE profiles in our expanded dataset revealed that all four samples of mbn2 cluster together as a single, well-supported group that is most similar to a cluster containing S2 cells (Figure 2E). The detailed relationships among sublines within the mbn2 cluster deviate slightly from expectations based on cell line history (Figure 2D); however, this discrepancy appears to be caused by differences in read length or coverage between the data from modENCODE and our study (Supplementary Figure S5). All mbn2 samples have the low SNP heterozygosity across most of their genomes that is characteristic of Schneider cell lines, and also share the small patch of heterozygosity at the base of chromosome arm 2L found in S2 and S2R+ cells (Supplementary Figure S3B). Additionally, all four mbn2 samples share widespread segmental aneuploidy across the entire euchromatin that is a common hallmark of S2 and S2R+ cells, but not other *Drosophila* cell lines (Supplementary Figure S3C). Together, these data support the conclusions that multiple independent sublines of mbn2 cells all share a common origin and are likely to descend from a single divergent lineage of S2 cells. Based on these observations, we speculate that currently circulating mbn2 cells derive from a mislabeling or cross-contamination event with S2 cells in the Gateff lab that occurred prior to distribution to the Hultmark or Dorn labs (node 4; Figure 2D). This scenario is consistent with the facts that S2 cells were developed and widely distributed prior to the origin of mbn2 cells (Schneider 1972; Gateff *et al.* 1980) and that there was a 12-year gap between the initial report describing mbn2 cells and use in any subsequent publication (Gateff *et al.* 1980; Samakovlis *et al.* 1992).

The possibility that mbn2 is a divergent lineage of S2 is plausible given that both cell lines are thought to have a hemocyte-like cell type (Gateff *et al.* 1980; Cherbas *et al.* 2011; Luhur *et al.* 2019). Furthermore, it is known that different lineages of *bona fide* S2 cells vary substantially in their morphology and gene expression, some of which share properties with mbn2 cells (Samakovlis *et al.* 1992; Yanagawa *et al.* 1998; Cherbas *et al.* 2011; Supplementary Figure S6). Under phase-contrast microscopy, canonical S2 cells represented by the S2-DRSC subline are generally a mix of loosely adherent spherical cells and simple round flat cells. In contrast, live S2R+ cells can be characterized by many “phase dark” cells that attach to the growth substrate, which can flatten out to exhibit both polygonal and “fried egg” morphology. S2R+ cells that are loosely attached to the growth surface are generally spherical with fine cell protrusions. Like S2R+ cells, mbn2 cells are characterized by a mix of flattened phase dark cells that assume the polygonal and fried egg morphology, as well as loosely adhering spherical cells. However, loosely adherent mbn2 cells have a bigger diameter relative to S2-DRSC and S2R+ cells. Recognition of mbn2 as a potentially divergent S2 lineage suggests that there is more phenotypic diversity among different S2 lineages than previously thought.

### A subset of LTR retrotransposon families are sufficient to identify *Drosophila* cell lines

Our analysis has thus far provided evidence that TE insertion profiles of commonly used *Drosophila* cell lines based on whole-genome sequences can be used to cluster cell lines and uncover cases of cell line misidentification. However, for these results to form the foundation for a *Drosophila* cell line authentication protocol, it is necessary to show that a cell line sample can successfully be identified on the basis of its TE profile. Furthermore, it is important to explore if whole-genome data are required for TE-based cell line identification in *Drosophila* since the cost of WGS could preclude its routine application by many labs. Therefore, we next investigated whether a subset of *Drosophila* TE families could potentially be sufficient for *Drosophila* cell line identification, with the aim of guiding the development of a cost-effective targeted PCR-based enrichment protocol that could be used more widely by the research community.

**Figure 3:**
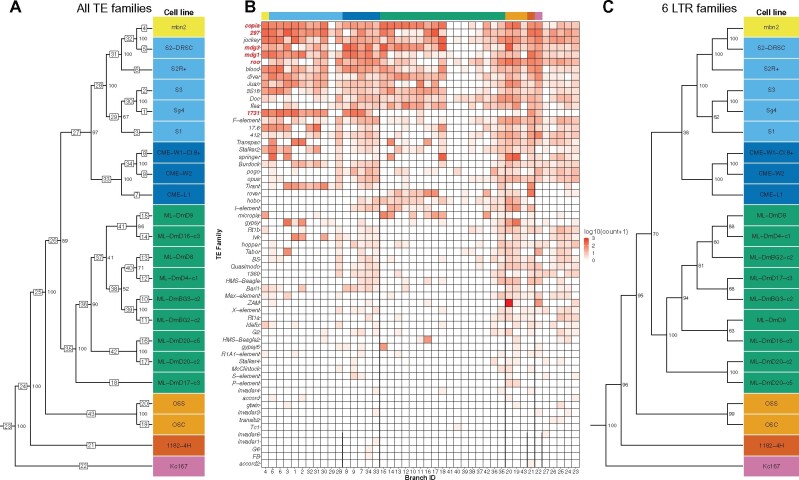
A small subset of LTR retrotransposon families can identify *Drosophila* cell lines. (A) Dollo parsimony tree of 22 *Drosophila* cell lines (without replicates) based on non-reference TE predictions for all 125 *D. melanogaster* TE families. Samples are colorized by lab origin as in Figure 2. Numbers inside boxes on branches indicate branch ID, and numbers beside nodes indicate percent support based on 100 bootstrap replicates. (B) Heatmap showing the number of non-reference TE insertion gain events per family on each branch of the tree in (A) based on ancestral state reconstruction using Dollo parsimony. The heatmap is colorized by log-transformed [log10(count + 1)] number of gains per family per branch, sorted top to bottom by overall non-reference TE insertion gains per family across all branches, and sorted left to right into clades representing lab origin with lab origin clade color codes indicated at the top of the heatmap. The six diagnostic LTR retrotransposon families used in (C) are highlighted in red. (C) Dollo parsimony tree of 22 *Drosophila* cell lines (without replicates) based on non-reference predictions of six LTR retrotransposon families (*297*, *copia*, *mdg3*, *mdg1*, *roo*, and *1731*). Numbers beside nodes indicate percent support based on 100 bootstrap replicates.

To investigate this possibility, we first clustered a non-redundant dataset of one “primary” replicate from each of the 22 *Drosophila* cell lines in the expanded dataset based on their whole-genome TE profiles (Figure 3A), which resulted in a similar clustering to the same sample of 22 cell lines including all replicates (Figure 2E). Replicates with the longest read length or depth of coverage were chosen as the primary replicate in the non-redundant dataset (Supplementary Table S1). We then took advantage of the ability of Dollo parsimony to reconstruct ancestral states and map the gain of TE insertions on each branch of the most parsimonious tree. TE insertions were then aggregated into families on each branch of the tree to visualize family- and branch-specific TE insertion profiles. This analysis revealed that a subset of 60 out of the 125 curated TE families in *D. melanogaster* are informative for *Drosophila* cell line clustering using TEMP predictions (Figure 3B, Supplementary File S3). Within the set of clustering-informative TE families, we observed that some TE families are broadly represented across many cell lines with different origins (*e.g.*, *copia*, *297*, *jockey*, *mdg3*, *mdg1*, and *roo*), although the quantitative abundance of these TE families varies across cell lines. Other TE families appear to be represented in only one cell line or a subset of cell lines from the same lab origin (*e.g.*, *ZAM*, *Tabor*, *HMS-Beagle2*, *gypsy5*, *1731*, *17.6*, *springer*, *Tirant*, *rover*, and *micropia*). These results provide systematic genome-wide evidence for the classical observation that proliferation of different TE families in cultured *Drosophila* cells is cell-line dependent (Echalier 1997). Additionally, these patterns of cell-line-specific TE proliferation provide further support for the conclusions that the DGRC Sg4 cell line is a lineage of S3 cells (all share *Ivk* proliferation), and that mbn2 cell lines are a divergent lineage of S2 cells (all share *1731* proliferation; Figure 3B).

Based on these results, we next evaluated whether a small, experimentally tractable subset of TE families is sufficient to cluster and identify *Drosophila* cell lines. For this analysis, we focused on LTR retrotransposon families since this type of TE inserts with intact termini and therefore provide reliable 5ʹ and 3ʹ junctions for targeted PCR-based enrichment protocols (Smukowski Heil *et al.* 2021). We used the pattern of family- and branch-specific TE insertion to heuristically guide the selection of a subset of six LTR retrotransposon families (*copia*, *297*, *mdg3*, *mdg1*, *roo*, and *1731*; TE family names highlighted in red in Figure3B), which defined unique TE profiles for each cell line and generated the same major patterns of *Drosophila* cell line clustering as the genome-wide dataset of all 125 TE families (Figure 3C). Finally, we tested whether a cell line sample (not used in the tree construction) can be accurately identified on the basis of its six-family TE profile. To do this, we used the six-family TE tree derived from the non-redundant set of primary replicates as a backbone to constrain Dollo parsimony searches including one additional “secondary” replicate for each of the 12 secondary replicates from the nine cell lines in the expanded dataset with secondary replicates. In 100% of cases (12/12), the additional secondary replicate clustered most closely with the primary replicate from the same cell line (Supplementary Figure S7). In 10/12 cases, the bootstrap support for the clustering of replicates was 100%, and the remaining two cases (both for CME-W1-Cl.8+) had lower bootstraps (≥64%) presumably because of the short-read length for these secondary replicates (50 bp). This proof-of-principle analysis indicates that TE insertions from a small subset of LTR retrotransposon families can accurately identify *Drosophila* cell line samples, and that only a subset of “diagnostic” TE families are needed to develop a *Drosophila* cell line authentication protocol. Based on these results, we have developed and validated a PCR enrichment-based NGS protocol that generates genome-wide TE profiles using this subset of LTR retrotransposon families and can be used to authenticate *Drosophila* cell lines at lower cost than WGS analysis (D. Mariyappa, D.B. Rusch, S. Han, A. Luhur, D. Overton, D.F.B. Miller, C.M. Bergman, A.C. Zelhof, unpublished results).

### TE profiles provide insight into *Drosophila* ovarian cell line history

The observation that different TE families are amplified in distinct *Drosophila* cell lines raises the question of whether a single TE family could diagnostically mark the identity of a *Drosophila* cell line or subline. One such candidate for this possibility is the retroviral-like LTR retrotransposon *ZAM* in the closely related OSS and OSC ovarian somatic cell lines (Niki *et al.* 2006; Saito *et al.* 2009). As shown above, we observed a massive increase in *ZAM* insertions in OSS cells relative to the OSC cell line (branches 19 and 20 in Figure 3, A and B), supporting previous findings by Sytnikova *et al.* (2014). However, Sytnikova *et al.* (2014) also reported that *ZAM* amplification did not occur in all OSS sublines, only in a contemporary subline of OSS cells (called OSS_C), but not in a putatively early passage subline of OSS cells (called OSS_E).

**Figure 4:**
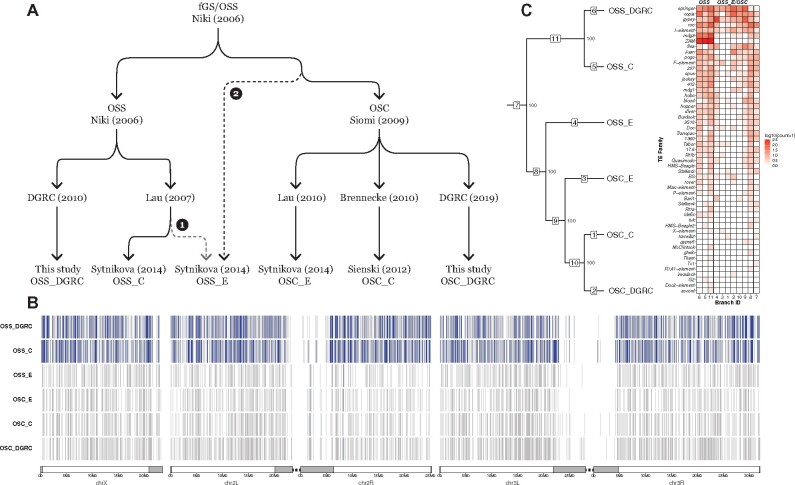
ZAM proliferation reveals OSS cell line identity. (A) Key events in the history of OSS and OSC cell line creation and distribution. Dotted lines represent alternative hypotheses for the identity of OSS_E. Branch 1 represents the reported provenance that hypothesizes OSS_E is an early-diverging OSS subline; branch 2 hypothesizes that OSS_E approximates an ancestral state of the OSC cell line. (B) Genome-wide non-reference TE insertion data for six ovarian cell lines with *ZAM* insertions highlighted in blue and all other TE families in gray. (C) Dollo parsimony tree of ovarian cell lines based on all non-reference TE predictions. Numbers inside boxes on branches indicate branch ID, and numbers beside nodes indicate percent support based on 100 bootstrap replicates. (Left) Heatmap showing the number of non-reference TE insertion gain events per family on each branch of the tree based on ancestral state reconstruction using Dollo parsimony. The heatmap is colorized by log-transformed [log10(count + 1)] number of gains per family per branch, sorted top to bottom by overall non-reference TE insertion gains per family across all branches and sorted left to right into the *bona fide* OSS and OSS_E/OSC clusters (right).

To address whether *ZAM* proliferation is restricted to a subset of OSS sublines or is in fact a specific marker for all OSS sublines, we performed an integrated analysis of TE predictions in WGS data from six OSS and OSC samples from our and two previous studies (Sienski *et al.* 2012; Sytnikova *et al.* 2014). To formulate alternative hypotheses and guide interpretation of our results, we first compiled the reported provenance of these six OSS and OSC cell line samples. As shown in Figure 4A, the ultimate ancestor of all OSS and OSC cell lines is a cell line composed of germline and somatic ovarian cell types called fGS/OSS (Niki *et al.* 2006). fGS/OSS cells were subsequently selected in the Niki lab to remove germline-marked stem cells to create the ancestor of the OSS (ovarian somatic sheet) cell line. The Niki lab sent two batches of OSS cells to the Lau lab in 2007 (N. Lau, personal communication): one was expanded and continuously cultured to become the OSS_C subline; the other was briefly cultured and stored as a cryopreserved culture for many years, then thawed and sequenced in 2013 creating the OSS_E sample (Sytnikova *et al.* 2014). Our sample of OSS cells comes from an independent subline donated by the Niki lab to the DGRC in 2010 (OSS_DGRC). The Niki lab also sent fGS/OSS cells to the Siomi lab, who independently selected against germline cells to create another somatic cell line called OSC (ovarian somatic cells; Saito *et al.* 2009). OSC cells were sent by the Siomi lab in 2010 separately to the Lau (OSC_C) and Brennecke (OSC_E) labs and were later donated by the Siomi lab to the DGRC in 2019 (OSC_DGRC).

Because WGS data from Sienski *et al.* (2012) and Sytnikova *et al.* (2014) is single-ended, integrated analysis of ovarian cell lines required a different TE prediction strategy than the one used for the analysis of the paired-end datasets above. Preliminary analyses revealed that some single-end TE predictors (*e.g.*, ngs_te_mapper, RelocaTE; Linheiro and Bergman 2012; Robb *et al.* 2013) severely under-predicted insertions specifically for the *ZAM* family in the DGRC OSS sample relative to TEMP results based on paired-end data (Supplementary Figure S8). Additionally, our analysis of OSS and OSC samples ultimately required tracking intra-sample TE allele frequencies, which is not available in other TE predictors that use single-end data (*e.g.*, TIDAL; Rahman *et al.* 2015). Thus, we developed a new implementation of the single-end TE predictor originally described in Linheiro and Bergman (2012) called ngs_te_mapper2 (https://github.com/bergmanlab/ngs_te_mapper2) that improves speed and sensitivity relative to the original version and has been extended to estimate intra-sample TE allele frequencies (Supplementary Figure S9 and Tables S4 and S5; see Supplementary Text for details).

Using datasets normalized to the same read length and coverage in order to optimize the resolution of closely related sublines, we predicted non-reference TE insertions in all OSS and OSC sublines with ngs_te_mapper2 (Supplementary File S4). These results revealed that *ZAM* has proliferated massively in the OSS_DGRC and OSS_C sublines (553 and 630 copies, respectively, in euchromatic regions), but is present in only one or two copies in OSS_E and all OSC sublines (Figure 4B). The abundance of *ZAM* in these ovarian cell lines is more than 10-fold higher than fly strains where *ZAM* has been mobilized because of deletions in the *flamenco* piRNA locus (Leblanc *et al.* 1999; Zanni *et al*. 2013) or because of multigenerational knockdown of the piRNA effector protein *piwi* (Barckmann *et al.* 2018; Mohamed *et al.* 2020).

Under the “reported provenance” hypothesis that OSS_E and OSS_C share a more recent common ancestor than they do with OSS_DGRC (branch 1; Figure 4A), this pattern of *ZAM* abundance can only be explained by unlikely scenarios such as a massive loss of *ZAM* insertions on the branch leading to OSS_E, or independent parallel amplifications of *ZAM* on the OSS_C and OSS_DGRC sublines. An alternative hypothesis to explain the pattern of *ZAM* abundance is motivated by another observation made by Sytnikova *et al.* (2014): OSS_E shares more TE insertions in common with OSC sublines (OSC_E and OSC_C) than it does with a contemporary OSS subline (OSS_C). This pattern is not expected under the reported provenance hypothesis and suggests that OSS_E may in fact be an OSC-like lineage, rather than an early passage OSS subline. Under this alternative “uncertain provenance” hypothesis (branch 2; Figure 4A), the only *bona fide* OSS sublines would be OSS_C and OSS_DGRC, and *ZAM* proliferation could truly be a diagnostic marker of OSS cell line identity.

To test these alternative hypotheses, we used ngs_te_mapper2 predictions as input to cluster OSS and OSC sublines using Dollo parsimony. We found two highly supported clusters, one containing only the OSS_C plus OSS_DGRC sublines and the other containing OSS_E plus all OSC sublines (Figure 4C, Supplementary File S5). Ancestral state reconstruction clearly demonstrated that high *ZAM* abundance is restricted to the cluster containing OSS_C and OSS_DGRC sublines. The only two *ZAM* insertions that are found in OSS_E and OSC sublines are both shared by multiple sublines and therefore likely inserted in a common ancestor of the entire clade (Figure 4B, Supplementary File S6). We verified that the clustering relationships among OSS and OSC sublines were not solely driven by the ZAM amplification by repeating our clustering analysis excluding *ZAM* insertions, obtaining the same topology as in the complete dataset (Supplementary Figure S11A).

**Figure 5:**
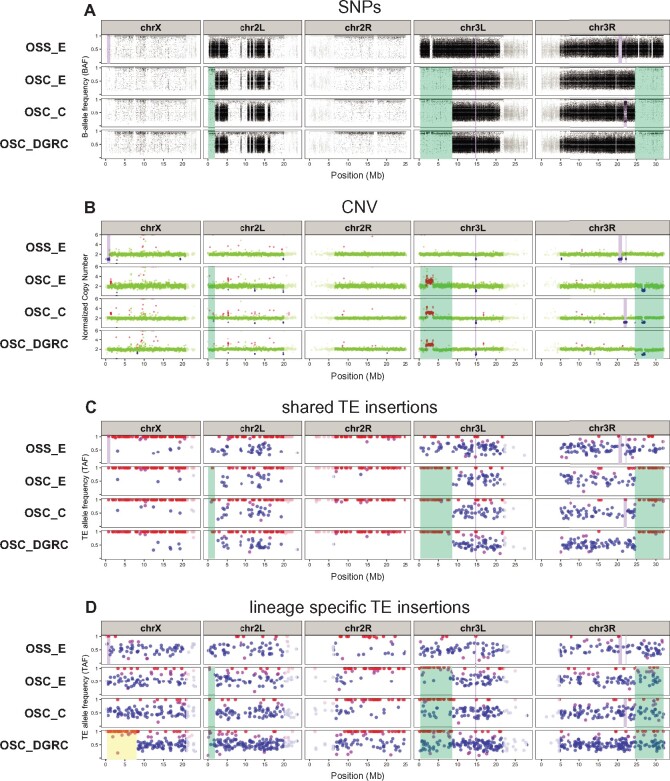
LOH, copy number evolution, and ongoing transposition shape TE profiles in *Drosophila* ovarian somatic cell lines. Genome-wide profiles for OSS_E and OSC sublines of (A) intra-sample allele frequency based on SNP variants, (B) copy number, (C) intra-sample allele frequency based on TE insertions shared by OSS_E and OSC sublines, and (D) intra-sample allele frequency based on lineage-specific TE insertions restricted to only OSS_E or the OSC sublines. SNPs and TE insertions in highly repetitive low recombination regions are shaded in grey. For SNP profiles, the BAF was determined as the coverage of reads supporting the non-reference allele divided by total coverage at that variant positions; regions of heterozygosity in a diploid genome are shown in BAF profiles where clusters of SNPs have allele frequencies centered around 0.5. For copy number profiles, each data point represents normalized copy number (ratio*ploidy) for a given 10-kb window estimated by Control-FREEC (Boeva *et al.* 2012); data points for each window are colorized by CNV status (red: CNV gain; green: no CNV; blue: CNV loss), which are based on the comparison between normalized copy number for that window and a baseline ploidy of 2*n*. For TE profiles, TE insertions are classified as being homozygous (red), heterozygous (blue), or undefined (purple) based on intra-sample allele frequencies estimated by ngs_te_mapper2. Green shading indicates copy-neutral LOH regions defined by more extensive patterns of SNP heterozygosity in OSS_E relative to OSC sublines that are putatively caused by mitotic recombination. Yellow shading indicates copy-neutral LOH regions based on runs of homozygous TE insertions in OSC_DGRC relative to other OSC sublines that are putatively caused by mitotic recombination. Purple shading indicates LOH regions that are putatively caused by segmental deletion.

Further support for the hypothesis that OSS_E is an OSC-like lineage can be found in patterns of SNP and CNV variation in these cell line genomes (Supplementary Figure S11, B and C). OSS_C and OSS_DGRC have essentially identical BAF profiles across the entire genome (Supplementary Figure S11B). In contrast, OSS_E and OSC sublines share a BAF profile everywhere but the distal regions on chromosome arms 2L, 3L, and 3R (Supplementary Figure S11B, Figure 5A). BAF profiles on all of chromosome X and arm 2R clearly differentiate OSS_C and OSS_DGRC (heterozygous) from OSS_E and OSC sublines (homozygous; Supplementary Figure S11B). Likewise, CNV profiles support the clustering of OSS_C with OSS_DGRC, and OSS_E with the OSC sublines. OSS_C and OSS_DGRC share a large deletion on chromosome X not found in OSS_E plus OSC sublines, and OSS_E plus the OSC sublines share a smaller deletion on chromosome arm 3L not found in OSS_C or OSS_DGRC (Supplementary Figure S11C). Based on these results, we conclude that OSS_E is a divergent lineage of OSC cells rather than early passage OSS cells, that *ZAM* amplification truly marks *bona fide* OSS cell lines (include the OSS line distributed by the DGRC), and that ngs_te_mapper2 TE predictions based on single-end WGS data can be effectively used to cluster *Drosophila* cell lines and reveal aspects of cell line history.

### Loss of heterozygosity impacts TE profiles in *Drosophila* cell culture

Reinterpreting OSS_E as a divergent lineage of OSC cells requires explaining both the similarity and distinctness of its TE, BAF, and CNV profiles from other OSC sublines. Two observations led us to hypothesize that OSS_E approximates an ancestral state of current OSC sublines. First, OSS_E occupies a basal position in the OSS_E plus OSC cluster based on TE profiles (Figure 4C). Second, the BAF profile for OSS_E shows heterozygosity that extends in the distal regions of chromosome arms 2L, 3L, and 3R relative to OSC sublines (green shading, Figure 5A). We propose that differences in BAF profiles in these distal regions are caused by LOH that occurred in an ancestor of all OSC sublines after divergence from the lineage leading to OSS_E. We infer that these large-scale distal LOH events were caused by a copy-neutral process such as mitotic recombination, since the baseline copy number in these distal LOH regions is the same in OSS_E and OSC sublines (Figure 5B, Supplementary Figure S11C). Similar to previous reports in a primate cell line (Osada *et al*. 2014), we do observe smaller copy number gain and loss events, respectively, within the large regions of LOH on chromosome arms 2L and 3R. However, these copy number events are much smaller and fully contained within the larger LOH regions and therefore unlikely to be the cause of the large-scale distal LOH events. Despite previous evidence for putatively polyploid cells in the OSC_E subline (Sytnikova *et al.* 2014), we infer a diploid baseline copy number in the “stem line” that leads to the majority of inherited cells in the OSS_E and OSC sublines, since BAF profiles (Figure 5A) and DNA density profiles (Supplementary Figure S10) of the bulk samples show modal values that together are consistent with diploidy but not any higher ploidy values.

If this evolutionary scenario is correct, shared TEs (which inserted prior to the divergence of OSS_E and OSC sublines) that are heterozygous in OSS_E are predicted to be homozygous in OSC sublines in distal LOH regions, but should maintain heterozygosity elsewhere in the genome. To test these predictions, we used intra-sample allele frequency estimates from ngs_te_mapper2 to classify the zygosity of TE insertions in OSS_E and OSC sublines. Evaluation of our classifier on simulated diploid genomes revealed it had >91% precision and crucially never falsely classified heterozygous insertions as homozygous (Supplementary Table S6), and is thus conservative with respect to detection of LOH using TE insertions. As predicted under our model, we observed that there are many shared TE insertions in distal LOH regions that are heterozygous in OSS_E but virtually all TE insertions in these regions are homozygous in OSC sublines (green shading, Figure 5C). Outside of distal LOH regions, shared TE insertions that are heterozygous in OSS_E generally retain heterozygosity in OSC sublines (Figure 5C). In contrast, we observe that many lineage-specific TE insertions (which occurred after the divergence of OSS_E and OSC sublines) are heterozygous in OSC sublines in distal LOH regions (green shading, Figure 5D). Together these results support the inferences that OSS_E approximates an ancestral state of current OSC sublines, that LOH events can cause fixation of previously heterozygous TE insertions in *Drosophila* cell lines, and that ongoing transposition in *Drosophila* cell culture can restore genetic variation in regions where previous large-scale LOH events have eliminated ancestral SNP or TE insertion variation.

Contrasting patterns of genetic variation between OSS_E and OSC sublines in distal regions of chromosome arms 2L, 3L, and 3R provided the initial evidence for LOH as mechanism of genome evolution in *Drosophila* cell culture. Additional evidence for copy-neutral LOH in *Drosophila* cell culture can be found in the lack of SNP heterozygosity on all of chromosome X and arm 2R (Figure 5A), which can be explained by whole-arm LOH events caused by centromere-proximal somatic recombination events in the common ancestor of the OSS_E/OSC lineage, assuming that the genome-wide heterozygosity observed in *bona fide* OSS sublines is ancestral (Supplementary Figure S11B). Consistent with the prediction of whole-arm LOH in the ancestor of the OSS_E/OSC lineage followed by ongoing transposition in cell culture, we observe that most shared TE insertions on chromosome X and arm 2R are homozygous (Figure 5C), while lineage-specific TE insertions are heterozygous (Figure 5D). Intriguingly, and in contrast to other OSC sublines, we also observe that lineage-specific TE insertions on the distal eight megabases of chromosome X in OSC_DGRC are almost all homozygous (yellow shading, Figure 5D). This observation can be explained by a secondary copy-neutral LOH event in the distal region of chromosome X that occurred recently only in the OSC_DGRC lineage. In this case, heterozygosity restored by ongoing TE insertion in *Drosophila* cell culture allows detection of subsequent LOH events in the same genomic region that cannot be detected using SNP variation.

In addition to large-scale LOH events affecting distal regions or whole chromosome arms that can be explained by copy-neutral processes such as mitotic recombination, we also observed smaller-scale LOH events that can be explained by hemizygosity due to segmental deletion (purple shading, Figure 5, A and B). For example, we observe a 200-kb region on chromosome arm 3L in all OSS_E and OSC sublines that lacks heterozygous SNPs which can be explained by a segmental deletion that that occurred in the common ancestor of the OSS_E/OSC lineage (Figure 5, Supplementary Figure S12). LOH by segmental deletion is supported by shared TEs in this region being homozygous in all OSS_E and OSC sublines. Likewise, in OSS_E, we observe two subline-specific segmental deletions on chromosome arm 3R of 900 and 100 kb, respectively that lack heterozygous SNPs and TE insertions in the corresponding regions (Figure 5, Supplementary Figure S12). We also observe a subline-specific segmental deletion on chromosome arm 3R of 800 kb in OSC_C that exhibits a BAF profile enriched at 0.85 (Figure 5, Supplementary Figure S12) rather than the homozygosity expected for complete LOH due to hemizygosity. Similar to patterns of LOH in mammalian tumors that have incomplete purity (Song *et al.* 2012), we interpret the incomplete LOH in this region as being caused by clonal heterogeneity in the OSC_C subline, with the majority of cells having the segmental deletion but a small proportion of cells lacking it. If this hypothesis is correct, the median copy number should be slightly over 1 in the segmentally deleted LOH region in OSC_C: as predicted, the median copy number of OSC_C in the putative LOH region is 1.14 (Supplementary Figure S12). Additionally, the OSS_E subline also exhibits a terminal deletion on the tip of chromosome X (Figure 5, Supplementary Figure S12), which does not lead to LOH in the SNP profile because of the primary whole-arm LOH event proposed to have occurred in the ancestor of the OSS_E/OSC lineage. However, similar to the secondary LOH event proposed to have occurred by somatic recombination on the distal region of chromosome X in OSC_DGRC, recovery of heterozygosity by ongoing TE insertion allows secondary LOH by segmental deletion to be observed in the lineage-specific TE-allele frequency (TAF) profile at the tip of chromosome X in OSS_E. Finally, we note that LOH events that can be explained by segmental deletions provide further support for a diploid stem line in the OSS_E/OSC lineage, since hemizygosity in a diploid is more parsimonious than scenarios such as multiple identical deletions or deletions followed by mitotic recombination required to explain LOH by segmental deletion in genomes with higher ploidies.

As LOH has not previously been reported as a mechanism of genome evolution in *Drosophila* cell culture, we sought to find additional evidence for this process by inspecting BAF profiles for other *Drosophila* cell lines in the expanded dataset. This led us to additional evidence for large-scale LOH events defined by SNPs on chromosome arms 2R and 3L of the CME-W2 and CME-W1-Cl.8+ cell lines (Supplementary Figures S3B and S13A), both of which are reported to have a diploid baseline autosomal copy number (Lee *et al.* 2014). As with OSS_E, we propose that the more extensive heterozygous BAF profile on these chromosome arms in CME-W2 represents the pre-LOH ancestral-like state, and the homozygous BAF profile of CME-W1-Cl.8+ represents the post-LOH derived state. This scenario is consistent with the reported establishment of CME-W1-Cl.8+ from a single cloned cell of a polyclonal cell line (CME-W1) with the same ancestral genotype as CME-W2 (Currie *et al.* 1988; Peel and Milner 1990). The lack of difference in the baseline copy number profiles on chromosome arms 2R and 3L of CME-W2 and CME-W1-Cl.8+ suggests these large-scale LOH events were also due to mitotic recombination (Supplementary Figure S13B). As predicted under the LOH model, we observed many TE insertions shared by CME-W2 and CME-W1-Cl.8+ are heterozygous in CME-W2 but are nearly all homozygous in CME-W1-Cl.8+ in LOH regions (Supplementary Figure S13C). Like in OSC sublines, we also observed many heterozygous TE insertions that are specific to CME-W1-Cl.8+ in LOH regions (Supplementary Figure S13D), consistent with recovery of TE insertion variation after LOH. Similar to the OSS_E/OSC lineage, we also find evidence in the CME-W2/CME-W1-Cl.8+ lineage for smaller-scale LOH events on chromosome arm 3R that can be explained by segmental deletion (Supplementary Figures S13 and S14), with clonal heterogeneity explaining incomplete LOH by segmental deletion at the tip of chromosome arm 3R in CME-W2. Finding evidence for both mechanisms of LOH in distinct cell lineages developed in different labs generalizes the inference that LOH shapes TE profiles in *Drosophila* cell lines and suggests that LOH in *Drosophila* culture is not dependent on the genetic background of the ancestral fly donor.

## Conclusions

**Figure 6:**
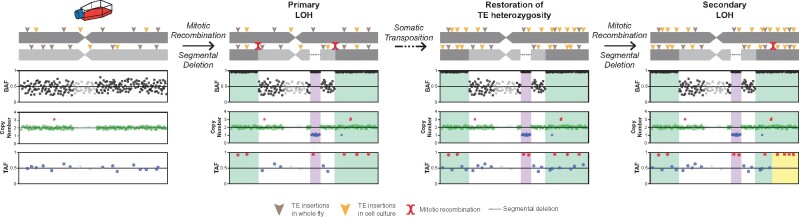
Schematic model of how LOH and somatic transposition interact to shape TE profiles in diploid *Drosophila* cell line genomes. Mitotic recombination (green shading) or segmental deletion (purple shading) can cause LOH of pre-existing heterozygous SNP and TE variants, as revealed by changes in BAF and TAF profiles. Ongoing transposition in cell culture leads to the accumulation of new haplotype-specific heterozygous TE insertions inside and outside of primary LOH regions. Recovery of TE heterozygosity allows detection of secondary LOH events (yellow shading) in regions of the genome that have previously undergone primary LOH events. Secondary LOH can occur by either mitotic recombination or segmental deletion, but only mitotic recombination is depicted here. We note that this model depicts a simplified case of diploidy which applies to some cell lines such as the OSS_E/OSC and CME-W2/CME-W1-Cl.8+ lineages, however, many cell culture genomes can have complex genome structure due to polyploidy and aneuploidy.

Here, we demonstrate that TE insertion profiles can successfully identify *Drosophila* cell lines and use this finding to clarify several aspects of cell line provenance in *Drosophila*. The success of this approach validates our basic model for how the joint processes of germline transposition in whole flies and somatic transposition in cell culture create TE profiles that uniquely mark *Drosophila* cell lines (Figure 1). We also show that TE insertion profiles can shed light on the evolutionary history of *Drosophila* cell lines derived from a common ancestral cell line and that LOH is an additional mechanism of genome evolution in cell culture that adds complexity to our basic model (Figure 6). During cell culture, LOH resulting from mitotic recombination (green shading) or segmental deletion (purple shading) purges ancestral variation and causes previously heterozygous SNPs and TE insertions to become fixed or lost within a cell line genome. Ongoing transposition in cell culture leads to the relatively rapid recovery of TE but not SNP heterozygosity, allowing secondary LOH events to be identified using TE insertions in regions that have previous lost ancestral variation due to primary LOH events (yellow shading). The emerging model of TE evolution in cell culture motivated by results presented here has direct implications for the development of protocols for cell line identification in *Drosophila* and contributes to our general understanding of the mechanisms of genome evolution in cell lines derived from multicellular organisms.

## Data availability

Raw sequencing data generated in our study are available in the SRA under BioProject PRJNA689777. Supplementary material is available at figshare: https://doi.org/10.25386/genetics.14884977. Supplementary File S1 contains non-redundant bed files from McClintock runs using TEMP module on the expanded dataset including 34 *Drosophila* cell line samples (reference TEs, *INE-1* insertions, and TEs in low recombination regions excluded). Supplementary File S2 contains clustered TE profiles in the format of binary presence/absence data matrix including 34 *Drosophila* cell line samples (reference TEs, *INE-1* insertions, and TEs in low recombination regions excluded). Supplementary File S3 includes data matrix of the number of non-reference TE insertion gain events per family on each branch of the most parsimonious tree used for the heatmap in Figure 3B. Supplementary File S4 includes non-redundant bed files from McClintock runs using ngs_te_mapper2 module on the normalized OSS and OSC dataset. Supplementary File S5 includes clustered TE profiles in the format of binary presence/absence data matrix including six OSS and OSC cell line samples. Supplementary File S6 includes data matrix of the number of non-reference TE insertion gain events per family on each branch of the most parsimonious tree used for the heatmap in Figure 4C.
